# Allosteric folding correction of F508del and rare CFTR mutants by elexacaftor-tezacaftor-ivacaftor (Trikafta) combination

**DOI:** 10.1172/jci.insight.139983

**Published:** 2020-09-17

**Authors:** Guido Veit, Ariel Roldan, Mark A. Hancock, Dillon F. Da Fonte, Haijin Xu, Maytham Hussein, Saul Frenkiel, Elias Matouk, Tony Velkov, Gergely L. Lukacs

**Affiliations:** 1 Department of Physiology and; 2 SPR-MS Facility, McGill University, Montréal, Quebec, Canada.; 3 Department of Pharmacology & Therapeutics, School of Biomedical Sciences, Faculty of Medicine, Dentistry and Health Sciences, The University of Melbourne, Melbourne, Australia.; 4 Department of Otolaryngology-Head and Neck Surgery,; 5 Adult Cystic Fibrosis Clinic, Montreal Chest Institute, and; 6 Department of Biochemistry, McGill University, Montréal, Quebec, Canada.

**Keywords:** Cell Biology, Pulmonology, Chloride channels, Drug therapy, Epithelial transport of ions and water

## Abstract

Based on its clinical benefits, Trikafta — the combination of folding correctors VX-661 (tezacaftor), VX-445 (elexacaftor), and the gating potentiator VX-770 (ivacaftor) — was FDA approved for treatment of patients with cystic fibrosis (CF) carrying deletion of phenylalanine at position 508 (F508del) of the CF transmembrane conductance regulator (*CFTR*) on at least 1 allele. Neither the mechanism of action of VX-445 nor the susceptibility of rare CF folding mutants to Trikafta are known. Here, we show that, in human bronchial epithelial cells, VX-445 synergistically restores F508del-CFTR processing in combination with type I or II correctors that target the nucleotide binding domain 1 (NBD1) membrane spanning domains (MSDs) interface and NBD2, respectively, consistent with a type III corrector mechanism. This inference was supported by the VX-445 binding to and unfolding suppression of the isolated F508del-NBD1 of CFTR. The VX-661 plus VX-445 treatment restored F508del-CFTR chloride channel function in the presence of VX-770 to approximately 62% of WT CFTR in homozygous nasal epithelia. Substantial rescue of rare misprocessing mutations (S13F, R31C, G85E, E92K, V520F, M1101K, and N1303K), confined to MSD1, MSD2, NBD1, and NBD2 of CFTR, was also observed in airway epithelia, suggesting an allosteric correction mechanism and the possible application of Trikafta for patients with rare misfolding mutants of CFTR.

## Abstract 2

Trikafta, the combination of type I corrector VX-661, type III corrector VX-445, and the potentiator VX-770, may be applied for various CFTR folding mutants.

## Introduction

Cystic fibrosis (CF), one of the most common lethal autosomal-recessive diseases in the White population, is a multiorgan disease caused by loss of function of the CF transmembrane conductance regulator (CFTR), an anion channel expressed at the apical membrane of secretory epithelia (1). Although a considerable fraction of ~2000 mutations in the *CFTR* gene causes CF (Cystic Fibrosis Mutation Database, http://www.genet.sickkids.on.ca/app), their theratyping (i.e., matching medications with mutations) remains incomplete (CFTR2 project, http://cftr2.org) (2).

Mutant CFTRs can be classified in 6 main classes according to their cellular phenotypes, characterized by expression (class I), folding (class II), gating (class III), conductance (class IV), quantity (class V), and peripheral stability (class VI) defects (3, 4). Many mutations are associated with multiple cellular defects, a phenomenon that served as the foundation for their recently proposed combinatorial classification, which also facilitates more effective theratyping efforts (5).

CFTR, a member of the ATP-binding cassette transporter C class (ABCC) family, comprises 2 homologous halves, each containing a hexa-helical membrane-spanning domain (MSD1 and MSD2) and a nucleotide-binding domain (NBD1 and NBD2) that are connected by the intrinsically disordered regulatory domain (RD) (6). The archetypal folding mutation, deletion of phenylalanine at position 508 (F508del), is present in about 80% of CF patients (1, 7, 8). F508del impairs the NBD1 stability and the coupled or cooperative domain folding of the channel (9–11). This leads to a combination of defects, consisting of severely impaired processing, plasma membrane (PM) expression, and stability, as well as defective gating of the F508del-CFTR (12–14).

In recent years, efforts have been focused on the development of small molecule CFTR modulators (CF drug development pipeline; http://www.cff.org/trials/pipeline), culminating in the recent FDA approval of Trikafta (Vertex Pharmaceuticals), the combination of the correctors VX-661 (tezacaftor) plus VX-445 (elexacaftor) and the gating potentiator VX-770 (ivacaftor), for treatment of patients with at least 1 F508del allele. Trikafta confers a gain in lung function of 10.0 percentage predicted forced expiratory volume in 1 second (ppFEV1) and 13.8 ppFEV1 in homozygous (15) and compound heterozygous (16) F508del patients, respectively, and it results in reduced pulmonary exacerbations and improved patient-reported quality-of-life scores (16). While the rescue efficacy of F508del-CFTR processing defect by the VX-661 plus VX-445 corrector combination has, in part, been demonstrated in human bronchial epithelia (17), the mechanism of action of VX-445 and its utility for correcting rare folding mutants remain elusive.

Based on the CFTR cooperative domain folding that is posttranslationally completed (11), the coupled domain misfolding of the F508del and other CF-causing missense mutations, as well as their susceptibility to genetic and pharmacological rescue, it was suggested that the correction of distinct folding defects is required to robustly restore the mutant channel folding (18–20). Furthermore, localized stabilization of CFTR domains by correctors (or pharmacochaperones) may be propagated to distant regions of the channel; thereby, corrector combinations may synergistically stabilize a variety of mutant proteins (21). This hypothesis gained traction by demonstrating that rescuing the structural defects of the NBD1-MSDs and NBD2-domain interfaces — as well as the NBD1 stability by type I, II, and III correctors, respectively — reinstated folding for not only the F508del-CFTR (18, 19, 21), but also a subset of rare folding mutants located in the MSD1, MSD2, and NBD2 domains (21).

Building on these observations and the profoundly improved F508del-CFTR biosynthetic processing by combining VX-445 with the type I corrector VX-661 (17), it was plausible to assume that VX-445 may represent a type II or III corrector. Here, we report that VX-445 can suppress the F508del-NBD1 misfolding in vitro. Since VX-445 also synergizes with class I and II correctors in rescuing the processing defect of both F508del-CFTR and rare folding mutants in a human bronchial cell model and primary human nasal epithelia (HNE), these findings suggest that VX-445 is potentially the first FDA-approved type III corrector with possible utility for a wide range of folding mutants.

## Results

### Synergistic rescue of F508del-CFTR misprocessing with VX-445 and type I and II correctors.

**Figure 1:**
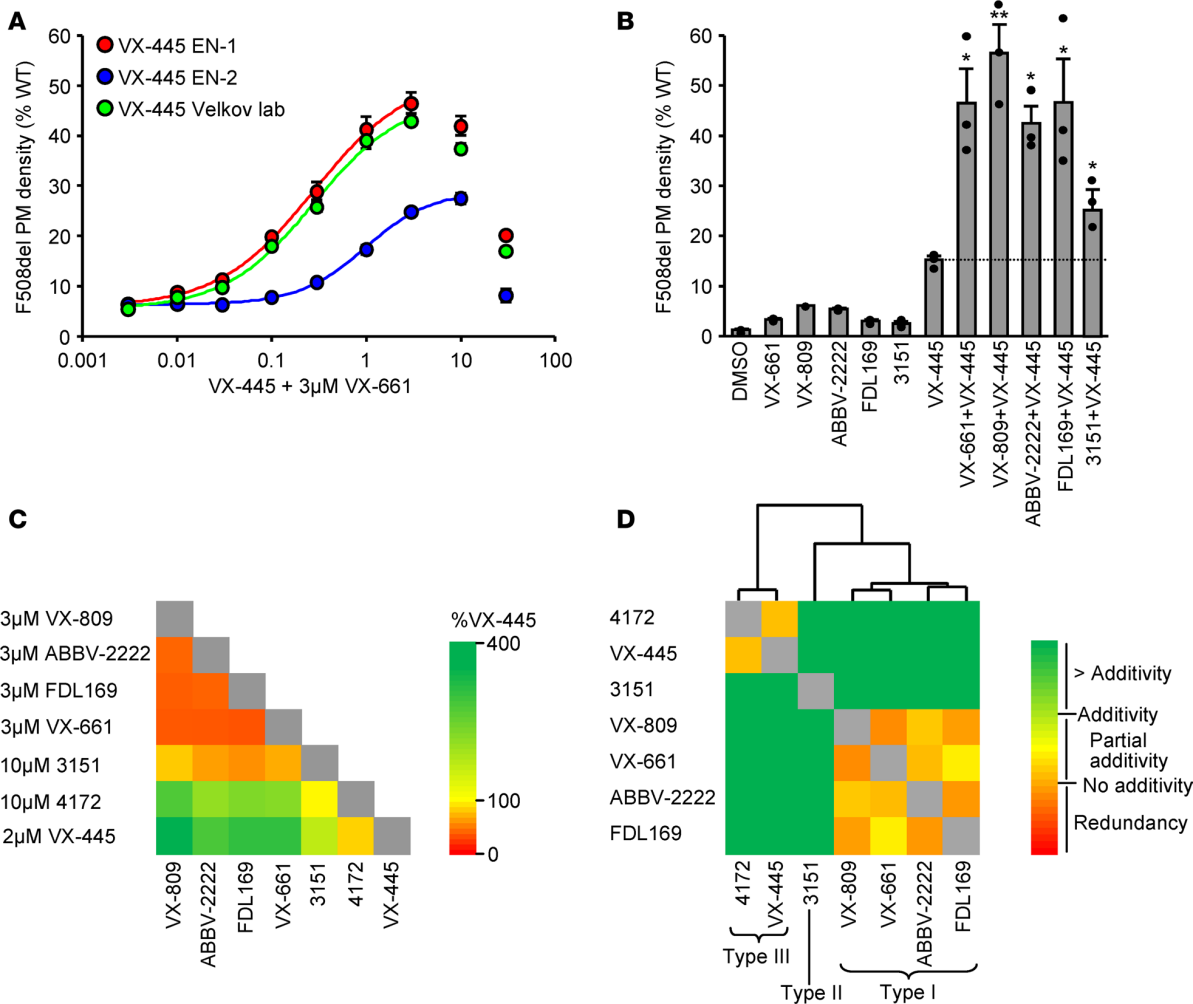
VX-445–mediated F508del correction is synergistic with type I and II correctors. (**A**) Dose-response of VX-445 (24 hours, 37°C) in presence of 3 μM VX-661 for the correction of F508del-CFTR PM density in CFBE41o- cells expressed as percentage of the WT-CFTR (*n* = 3). EN1, enantiomer 1; EN2, enantiomer 2. (**B**) PM density of F508del-CFTR after type I corrector (VX-661, VX-809, ABBV-2222, or FDL169; 3 μM, 24 hours, 37°C), VX-445 (2 μM, 24 hours, 37˚C) or type I plus VX-445 corrector combination treatment expressed as percentage of WT-CFTR in CFBE41o- (*n* = 3). (**C**) Heatmap of the effect of corrector combinations on the PM density of F508del-CFTR expressed in CFBE41o- (*n* = 3). (**D**) Heatmap of the combinatorial profiling established by calculating the dual corrector effect in relation to the theoretical additivity of the compounds. Combinatorial profiles were subsequently used to cluster compounds by average linkage analysis, and the distance was determined by Spearman’s rank correlation. The underlying data are depicted as bar plots in Supplemental Figure 1B. Data in **A** and **B** are means ± SEM of 3 independent experiments. *P < 0.05, **P < 0.01, by 1-way ANOVA followed by Turkey’s post hoc test.

To elucidate its mechanism of action, VX-445 was synthesized according to the publicly available patent description (WO 2018/107100), and its structure was validated as described in Methods. Subsequently, VX-445 (VX-445 enantiomer 1; VX-445 EN1) and its enantiomer (VX-445 EN2) also became commercially available. To determine the potency and efficacy, the effect of VX-445 EN1 and the Velkov lab preparation were evaluated on the PM density of F508del-CFTR overexpressed in CFBE41o- cells, a bronchial epithelial cell line (22). In the presence of VX-661, both VX-445 preparations increased the PM density of F508del-CFTR to ~45% of the WT, with an EC_50_ of ~0.28 μM (Figure 1A). At concentrations of ≥ 10 μM, VX-445 decreased F508del-CFTR PM density without affecting the cell viability (Figure 1A). In contrast, VX-445 or VX-661 alone elevated the F508del-CFTR PM density to ~15% or 3.5% of the WT, respectively (Figure 1B). Interestingly, the enantiomer of VX-445 (VX-445 EN2), harboring a (4R)-methyl instead of (4S)-methyl in the trimethylpyrrolidin ring, exhibited ~50% reduced efficacy in presence of VX-661, rescuing ~25% of the WT PM density with an EC_50_ of ~1.1 μM (Figure 1A). For subsequent experiments, the Velkov lab preparation of VX-445 was used.

To assess whether synergistic correction of the F508del-CFTR could be achieved by other corrector combinations, VX-445 was tested in the presence of correctors that are approved or under clinical development. The stabilization of the MSD1 and the profolding action of the FDA-approved corrector VX-809, similar to VX-661 (23), requires the presence of amino acids 370–380 in CFTR, which constitute part of TM6 and its linker to the NBD1 (21, 24). Combinations of VX-809 and VX-661 do not exhibit additive rescue effect (Supplemental Figure 1B; supplemental material available online with this article; https://doi.org/10.1172/jci.insight.139983DS1). ABBV-2222, a corrector compound currently in phase II clinical trials (25), likewise requires the presence of amino acids 370–380 for corrector function (26), shows similar efficacy as VX-809 for F508del-CFTR correction with a EC_50_ of ~0.02 μM (Supplemental Figure 1A), and shows no additivity with VX-661 or VX-809 (Supplemental Figure 1B). FDL169, a CFTR corrector whose development was halted after a phase I clinical trial (https://www.cff.org/Trials/pipeline/details/10121/FDL169), exhibits ~60% of the efficacy of VX-809 for F508del correction with a EC_50_ of ~0.2 μM (Supplemental Figure 1A) and exhibits no additivity with VX-809 (Supplemental Figure 1B). Thus, VX-661, VX-809, ABBV-2222, and FDL169 exhibit a common type I corrector mechanism in relation to F508del-CFTR folding rescue. In combination with these type I correctors, the impact of the VX-445 is synergistic and increases the F508del-CFTR PM density to 42%–56% of the WT (Figure 1B). Combination of VX-445 with the investigational type II corrector 3151 (21) also increased the F508del-CFTR PM density to ~25% of the WT (Figure 1B).

### VX-445 mechanism of action.

To interrogate the VX-445 mechanism of action, first we tested the F508del-CFTR correction efficiency by pairing VX-445, type I correctors, the type II corrector 3151, and the type III corrector 4172. The rescue effect of corrector pairs on the F508del-CFTR PM density was determined at or near saturating concentrations, to minimize possible additivity of compounds competing for the same binding site, and the results were analyzed by combinatorial profiling. Similar to 4172, the efficacy of VX-445 was increased in the presence of type I or type II correctors (Figure 1C and Supplemental Figure 1B). Next, we compared the PM density gain of F508del-CFTR elicited by corrector pairs relative to that of the calculated additive effect. This allowed us to categorize the corrector combinations as redundant, partially additive, additive, or super additive (Figure 1D and Supplemental Figure 1B). Subsequent clustering of the combination profiles revealed correctors that show comparable interactions with other compounds and, thus, likely work by a similar mechanism or compete for an overlapping binding site. As expected, the type I correctors VX-661, VX-809, ABBV-2222, and FDL169 clustered together, while the type II corrector 3151 and the type III corrector 4172 resided in separate clusters. VX-445 exhibits additivity or synergy with all correctors except 4172. In light of these observations and VX-445 clustering with 4172, it is likely that VX-445 exerts a type III corrector mechanism.

**Figure 2:**
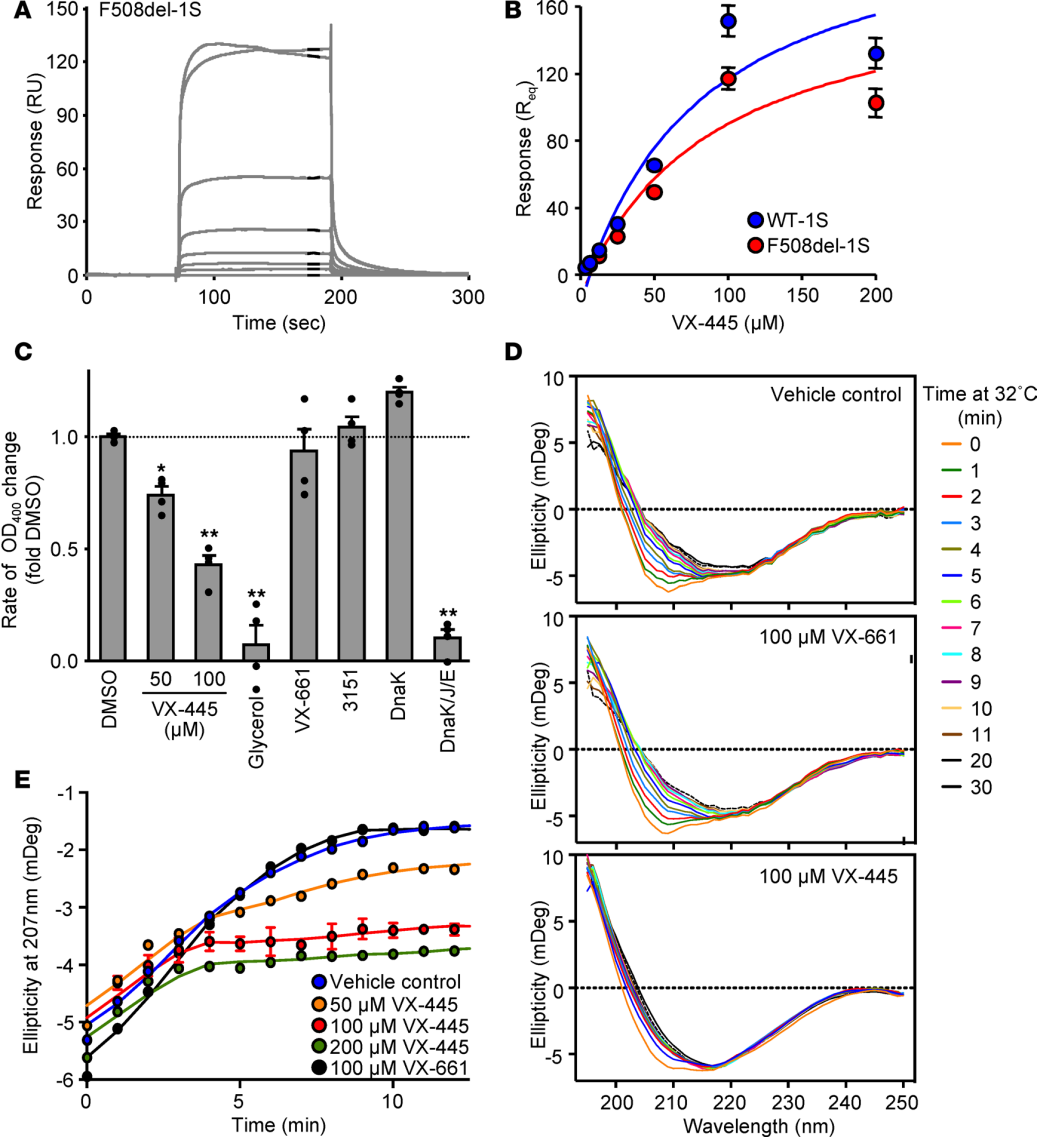
VX-445 binds to and changes the unfolding trajectory of CFTR-NBD1. (**A**) Representative surface plasmon resonance (SPR) sensorgram for the binding of VX-445 (0–200 μM) to immobilized F508del–NBD1-1S. (**B**) Binding isotherms for VX-445 binding to immobilized F508del–NBD1-1S or WT–NBD1-1S as determined by SPR (*n* = 3). Curve fitting was performed as described in Methods. (**C**) Aggregation rates observed in NBD1 aggregation assays between 30 and 50 minutes for the different compounds were normalized by the rate observed for F508del–NBD1-1S in 1% DMSO (*n* = 4). Compound/chaperone concentrations were 50 or 100 μM VX-445, 10% glycerol, 100 μM VX-661, 100 μM 3151, 10 μM DnaK, or DnaK-DnaJ-GrpE at 10, 2, and 10 μM, respectively. (**D**) Protein secondary structure stability was studied by far-UV CD spectra of F508del–NBD1-1S. CD scans between 250 and 195 nm were taken every minute at 32°C in the presence of vehicle control (1% 1,4-Dioxane), 100 μM VX-661, or 100 μM VX-445. CD scans obtained at different time intervals of 1 representative experiment were overlaid. (**E**) Quantification of the ellipticity values (in mDeg) observed at 207 nm. Values were plotted as a function of time in the presence of vehicle control (1% 1,4-Dioxane); 50, 100, or 200 μM VX-445; or 100 μM VX-661 (*n* = 2–3). Continuous lines were derived by 4-point smooth iteration. Data in **B**, **C**, and **E** are means ± SEM of the indicated number of independent experiments. *P < 0.05, **P < 0.01 by 1-way ANOVA followed by Turkey’s post hoc test.

To further characterize the VX-445 mechanism of action, we used the surface plasmon resonance (SPR) to test whether it could directly bind to the in vivo biotinylated F508del-NBD1 containing a single stabilizing mutation F494N (1S) (27). VX-445 displayed saturable, dose-dependent binding to immobilized F508del–NBD1-1S with a *K_D_* of ~80 μM, similar to the type III corrector 4172 (21) (Figure 2, A and B; Supplemental Figure 2; and Supplemental Table 1). Similar affinity was observed for VX-445 binding to WT–NBD1-1S, suggesting that its interaction is independent of the presence of the F508del mutation (Figure 2B). Although VX-661 bound to F508del–NBD1-1S, saturation of the binding sites was not reached up to 200 μM, suggesting a weak or unspecific interaction (Supplemental Figure 2B). This is in agreement with a previous report suggesting that type I correctors interact with NBD1 at high concentrations (28).

It has been established that the NBD1 thermal unfolding leads to the domain oligomerization and aggregation, which can be monitored by absorbance changes due to increased light scattering (29–31). To test whether VX-445 associates with F508del-NBD1 native-like conformer or its early unfolding intermediates, the compound’s capacity to suppress the aggregation of F508del–NBD1-1S was determined first. Time-dependent aggregation of the F508del–NBD1-1S was monitored at 32°C by light scattering at 400 nm (30, 32). VX-445, but not VX-661 or 3151, decreased the rate and the extent of F508del–NBD1-1S aggregation by 50% at 100 μM (Figure 2C and Supplemental Figure 2D). The aggregation of F508del–NBD1-1S was suppressed by the bacterial molecular chaperone and cochaperones complex (DnaK-DnaJ-GroE) and glycerol but not by DnaK alone (Figure 2C and Supplemental Figure 2D), similarly to the eukaryotic Hsc70/Hdj-2 but not Hsc70 alone (30). Thermal stabilization of NBD1 by second-site solubilizing and suppressor mutations (6SS, ΔRI/2PT/M470V/S495P/R555K) also prevented thermal aggregation (Supplemental Figure 2E). Remarkably, a partial loss of the F508del–NBD1-1S secondary structure was observed immediately after resuspending the domain at 32°C by measuring its ellipticity with circular dichroism (CD) spectroscopy (Figure 2D), implying that unfolding precedes the onset of the macroscopic aggregation, detected only at > 20 minutes of incubation (Supplemental Figure 2D). Importantly, VX-445 but not the type I corrector (VX-661) partially suppressed the F508del–NBD1-1S thermal ellipticity loss (Figure 2, D and E). These results jointly suggest that VX-445 binds to and changes the unfolding trajectory of the NBD1 domain, consistent with a type III corrector mechanism.

### Functional correction of human CFTR^F508del/F508del^ nasal epithelia.

**Figure 3:**
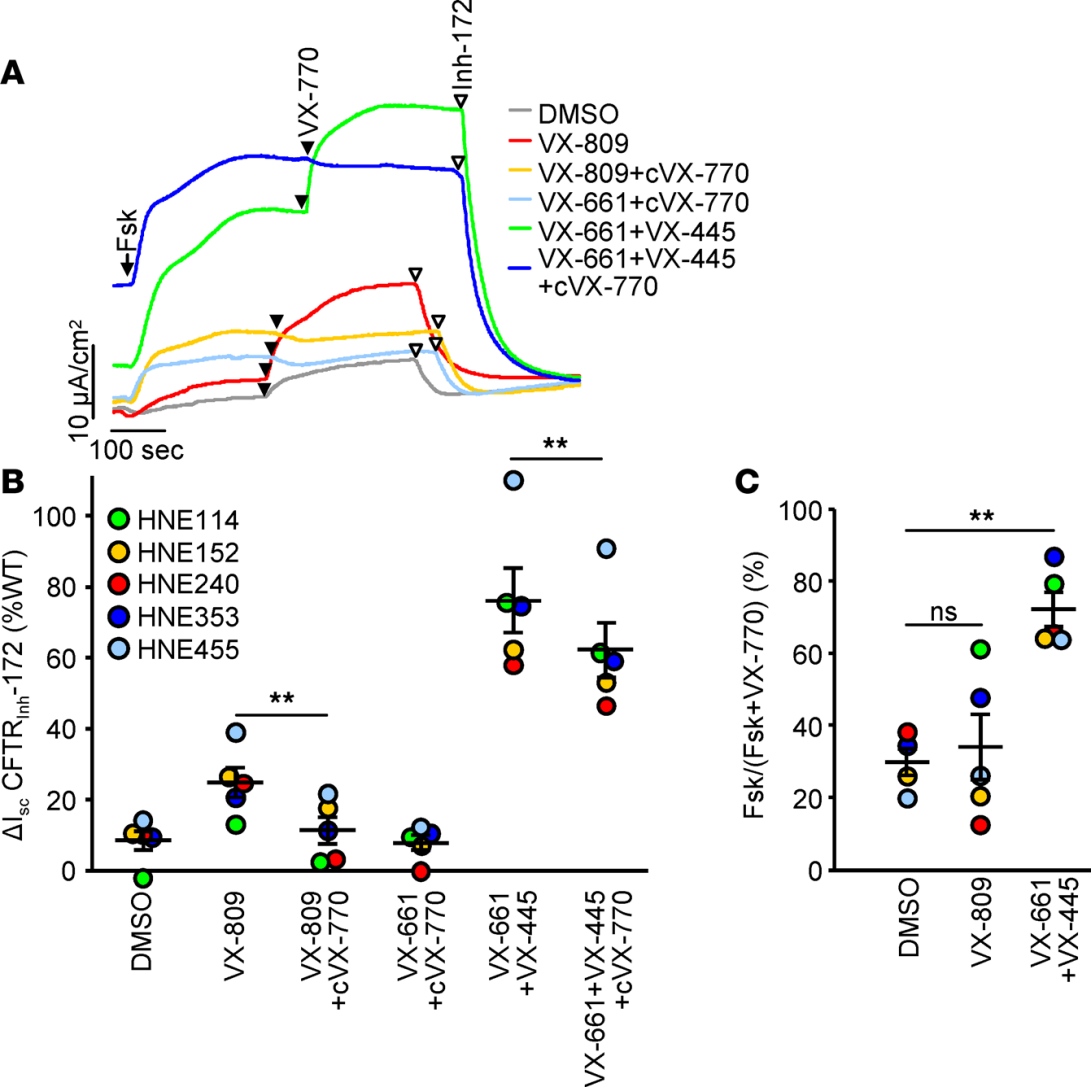
Trikafta mediated correction of *CFTR^F508del/F508del^* in HNE. (**A**) Effect of indicated single correctors or corrector combinations on the I_sc_ of human nasal epithelia with *CFTR^F508del/F508del^* genotype (CF-HNE). CFTR-mediated currents were induced by sequential acute addition of forskolin (Fsk, 20 μM, arrow) and VX-770 (770, 10 μM, filled arrowhead), followed by CFTR inhibition with CFTR_inh_-172 (Inh-172, 20 μM, open arrowhead) in intact monolayers with basolateral-to-apical chloride gradient. (**B**) Quantification of the CFTR_inh_-172 inhibited current, after stimulation as in **A**, in CF-HNE isolated from 5 different homozygous F508del CF patients after single correctors (VX-809 and VX-661, 3 μM; VX-445, 2 μM; 24 hours, 37°C), corrector plus chronic potentiator (cVX-770, 1 μM, 24 hours, 37°C) or corrector combination treatment expressed as percentage of WT-CFTR currents in WT-HNE from 10 donors. **P < 0.01 by paired 2-tailed Student’s *t* test. (**C**) The fraction of potentiator-independent (forskolin-induced) current in HNE treated with single corrector or corrector combination. **P < 0.01 by paired 2-tailed Student’s *t* test followed by Bonferroni’s FDR correction.

While the efficacy of the VX-661 plus VX-445 combination for the correction of F508del-CFTR in patient-derived respiratory epithelia has been demonstrated, the level of channel correction in relation to the WT-CFTR current remains unreported (17). We monitored the CFTR function in HNE isolated from 5 homozygous F508del patients (CF-HNE) and from 10 non-CF individuals without chronic inflammation (WT-HNE) after differentiation at air-liquid interface (ALI). VX-809 treatment increased the forskolin-activated and acutely VX-770–potentiated short-circuit current (I_sc_) of F508del-CFTR from 9% to 25%, relative to that of the WT (Figure 3, A and B, and Supplemental Figure 3) as reported before (21, 33). Chronic treatment with 1 μM VX-770, a concentration that is therapeutically reached in respiratory epithelia (34), was reported to significantly decrease the functional correction by VX-809 in the presence of acute potentiator addition (Figure 3, A and B), as observed in CF-HBE (35, 36) and CF-HNE (21). Biochemical analysis of the complex-glycosylated F508del-CFTR in CF-HBE corrected by VX-661 plus VX-445 also suggested downregulation upon chronic exposure to VX-770 (17).

VX-661 plus VX-445 treatment increased the F508del-CFTR current to 76% or 62% of the WT in the presence of acute or chronic VX-770 exposure, respectively (Figure 3, A and B). The magnitude of CFTR I_sc_ varied between 19 and 36 μA/cm^2^ in corrected CF-HNE and between 14 and 50 μA/cm^2^ in WT-HNE, isolated from different individuals. While the cause for the large variation of the CFTR I_sc_ is not known, values of the WT samples partly overlapped with the VX-661 plus VX-445 corrected F508del-CFTR I_sc_, suggesting that near WT-like channel activity could be achieved in F508del-CFTR HNE (Supplemental Figure 3). It is plausible that improved conformational rescue with VX-661 and VX-445 combination at least partly accounts for the attenuated gating defect of the F508del-CFTR, indicated by a 2-fold increase in the forskolin-induced, potentiator-independent fractional activation of the I_sc_ (Figure 3C), similar to that observed earlier for triple corrector combination (21).

### Allosteric rescue of rare CFTR folding mutants with VX-661 and VX-445.

**Figure 4:**
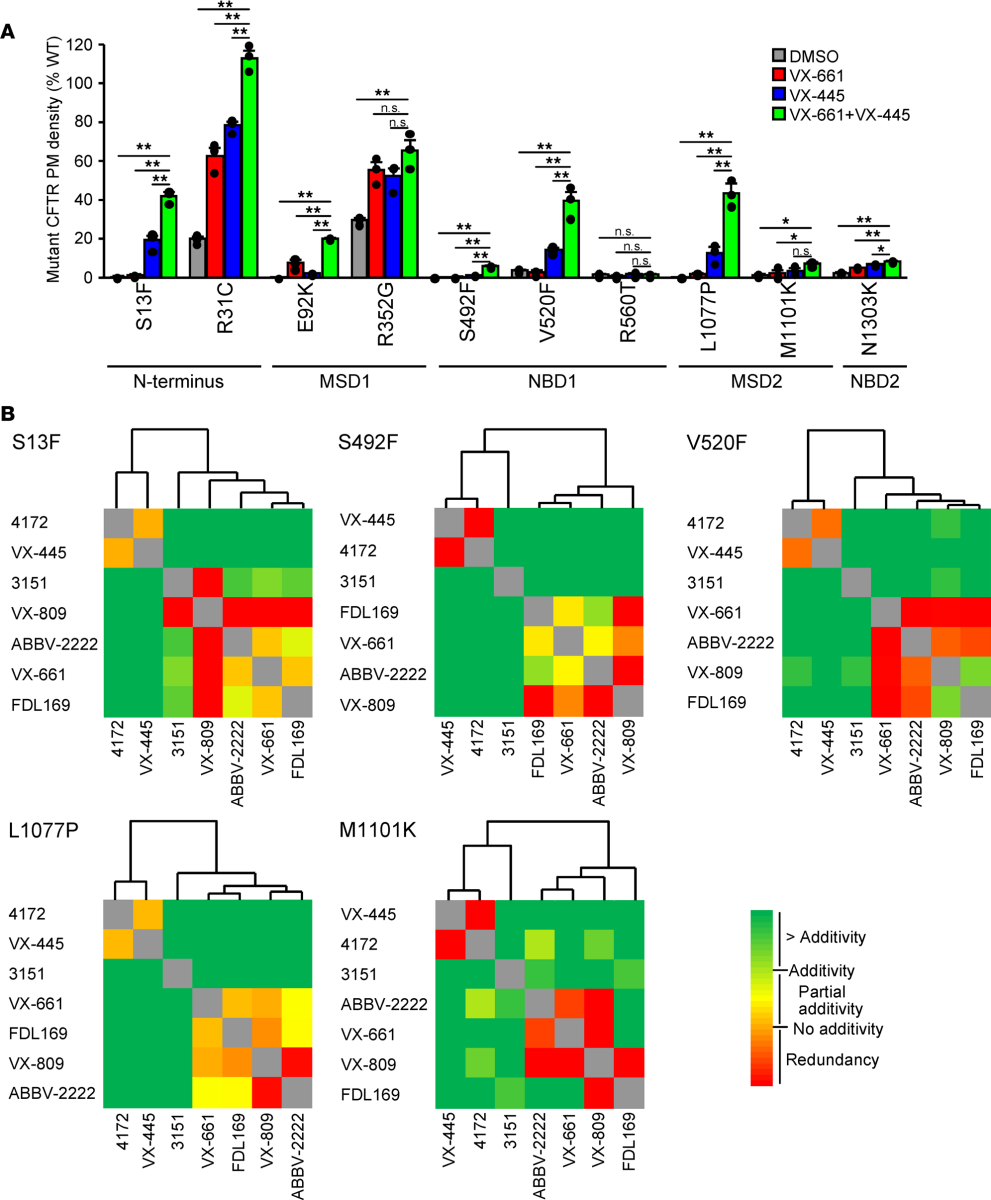
Efficacy, combinatorial profiling, and clustering of mechanistic classes of correctors in rare CFTR folding mutants. (**A**) PM density of the indicated CFTR mutants alone and after VX-661 (3 μM), VX-445 (2 μM), or combination treatment expressed as percentage of WT-CFTR in CFBE41o- (*n* = 3). Data are means ± SEM. *P < 0.05 and **P < 0.01 by 1-way ANOVA followed by Turkey’s post hoc test. (**B**) Heatmaps of the combinatorial profiling established by calculating the dual corrector effect in relation to the theoretical additivity of the compounds for S13F, S492F, V520F, L1077P, and M1101K-CFTR in CFBE41o-. Combinatorial profiles were subsequently used to cluster compounds by average linkage analysis, and the distance was determined by Spearman’s rank correlation. The underlying data are depicted as bar plots in Supplemental Figure 5, A–E.

We have shown that certain missense mutations, distributed throughout CFTR, manifest in propagated conformational defects in all structured domains of the channel (11), and this phenomenon was mitigated by allosteric preclinical folding correctors, independent of the mutation location (21). To test whether VX-661 plus VX-445 have a similar potential, we interrogated the rescue efficiency of 10 CFTR mutants that are distributed throughout the channel (Figure 4A) and are associated with folding and processing defects (5, 21, 37). All the mutations, except R31C and R352G, lead to > 95% reduction of the mutant protein PM density relative to the WT after normalization for mRNA levels (Figure 4A, Supplemental Figure 4, and Supplemental Figure 5). Four of these mutant proteins (S492F, V520F, R560T, and M1101K) were resistant to type I correctors (e.g., VX-661 and VX-809) (Figure 4A; Supplemental Figure 4, E and F; and Supplemental Figure 5, B and C). Remarkably, the PM density of 9 out of 10 mutant proteins was significantly increased by VX-661 plus VX-445 treatment, and in 8 mutant CFTRs, the response to this corrector combination was significantly larger in comparison with single correctors, reaching therapeutically significant levels of > 20% of WT in S13F-, R31C-, E92K-, R352G-, V520F-, and L1077P-CFTR (Figure 4A).

If allosteric correction of distinct folding defects of the mutants can be attributed to the corrector combination action, additivity or synergism between different type correctors would be expected. To test this assumption, combinatorial profiling of correction efficiency of rare CFTR folding mutants was performed. This analysis, however, was not possible for some mutant CFTRs for the following reasons: R560T did not respond to correctors, R31C and R352G yielded > 50% PM density upon single corrector treatment and displayed only partial additivity of corrector combinations, E92K exhibited > 3-fold higher response to ABBV-2222 in comparison with other type I correctors, and N1303K showed only partial additivity between different corrector types, despite low absolute correction (Supplemental Figure 4, B–F). In contrast, responses to some corrector combinations surpassed theoretical additivity for S13F-, S492F-, V520F-, and L1077P-CFTR (Supplemental Figure 5, A–E). Cluster analysis of the combination profiles in these mutant proteins and M1101K-CFTR, similar to that of F508del-CFTR, indicated 3 clusters of compounds; type I correctors VX-661, VX-809, ABBV-2222, and FDL169 clustered together; type III correctors 4172 and VX-445 formed a cluster; and the type II corrector 3151 formed its own category with variable distance to the other clusters (Figure 4B). These results reinforce the notion that combinations of different type of correctors, including VX-661 plus VX-445, simultaneously stabilize distinct domains and/or domain interfaces, resulting in improved cooperative domain folding of a variety of folding mutants, which would likely benefit patients carrying these mutations.

### Identification of rare folding mutants showing partial functional correction by VX-661 plus VX-445 in HNE.

**Figure 5:**
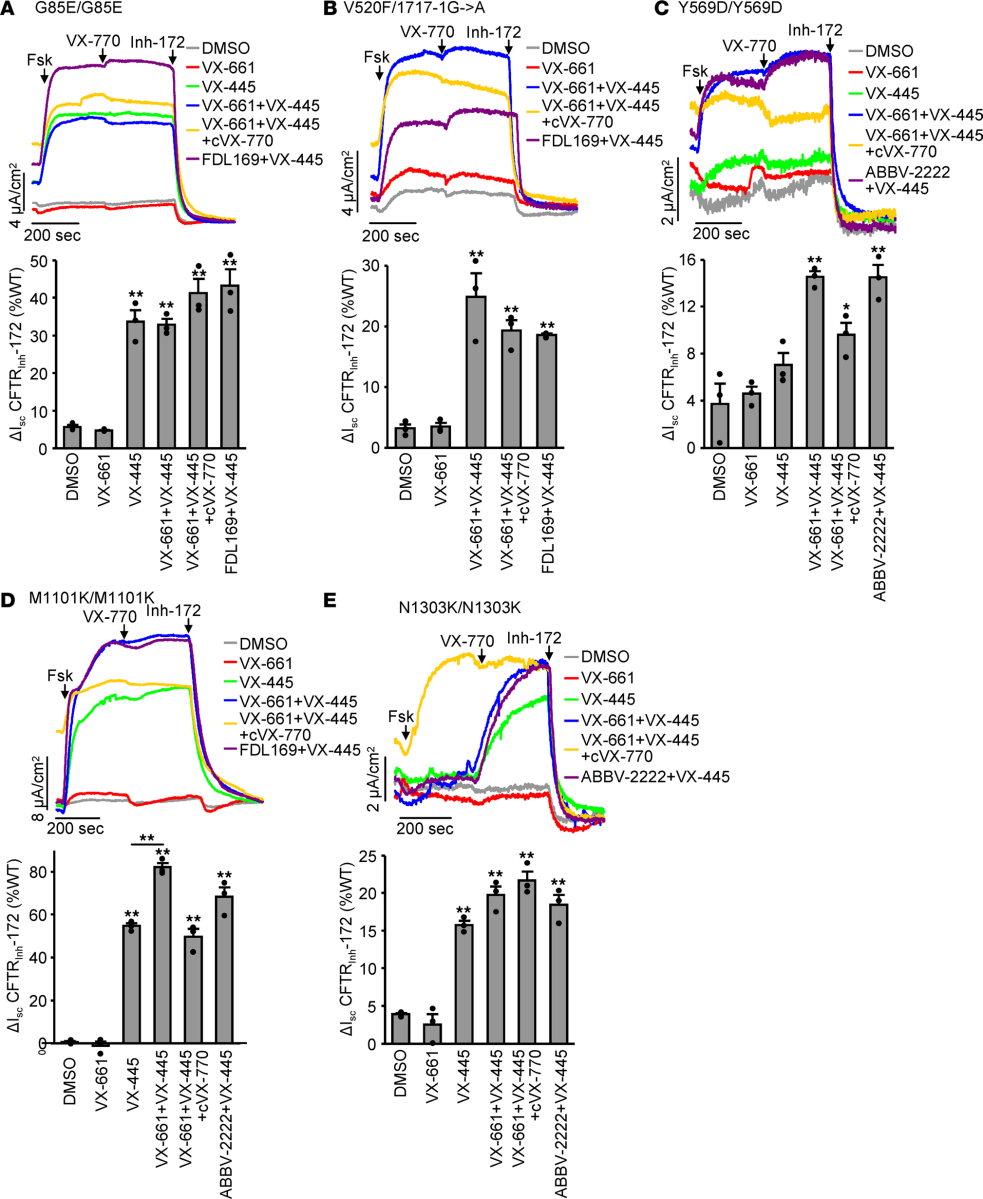
Efficacy of Trikafta for the functional correction of rare CFTR folding mutants in HNE. (**A–E**) Effect of indicated single correctors (VX-661, ABBV-2222, and FDL169, 3 μM; VX-445, 2 μM; 24 hours), corrector plus chronic potentiator (cVX-770, 1 μM, 24 hours), or corrector combinations on the I_sc_ of HNE with *CFTR*^G85E/G85E^ (**A**, *n* = 3), *CFTR*^V520F/1717-1G->A^ (**B**, *n* = 3), *CFTR^Y569D/Y569D^* (**C**, *n* = 3), *CFTR^M1101K/M1101K^* (**D**, *n* = 3), or *CFTR^N1303K/N1303K^* (**E**, *n* = 3) genotype. Representative traces (top panels) and quantification of the CFTR_inh_-172 inhibited current expressed as percentage of WT-CFTR currents in HNE from 10 donors (bottom panels). CFTR-mediated currents were induced by sequential acute addition of forskolin (Fsk, 20 μM) and VX-770 (10 μM) followed by CFTR inhibition with CFTR_inh_-172 (Inh-172, 20 μM) in an intact monolayer with basolateral-to-apical chloride gradient. Data are means ± SEM of 3 measurements. *P < 0.05 and **P < 0.01 by 1-way ANOVA followed by Turkey’s post hoc test.

Patient-derived HNE are precision medicine tools that can predict the clinical responsiveness of individuals and likely inform on the theratype of individual mutations or mutation combinations (38–40). To analyze the VX-661 plus VX-445 response on individual mutations rather than compound heterozygous genotypes, we used homozygous HNE with the mutations G85E, Y569D, M1101K, D1152H, or N1303K, as well as HNE with the folding mutation G85E or V520F on 1 allele and the splice mutation 1717-1G->A on the second allele, which likely does not give rise to correctable protein expression (2). With the exception of D1152H, these mutant CFTRs were associated with severely reduced channel function (< 5% of the WT), and treatment with VX-661 alone or in combination with acute VX-770–mediated potentiation failed to correct the mutant I_sc_ (Figure 5 and Supplemental Figure 6). VX-445 alone significantly increased G85E, M1101K, and N1303K-CFTR function, which was augmented by type I correctors (VX-661 or ABBV-2222) for M1101K-CFTR (Figure 5, A, D, and E). While Y569D was unresponsive to VX-445 alone, this mutant protein and V520F-CFTR were responsive to VX-445 plus type I corrector combinations (Figure 5, B and C).

We reported before that chronic exposure to potentiators can attenuate the corrector efficacy in some rare CFTR mutants (33). Here, we observed downregulation of the VX-661 plus VX-445 corrected maximal current in Y569D and M1101K-CFTR by chronic VX-770 treatment (Figure 5, C and D). Nevertheless, the VX-661 plus VX-445 corrected I_sc_ of heterozygous V520F, homozygous G85E, M1101K, and N1303K-CFTR reached 20%, 41%, 50%, and 22% of the WT I_sc_, respectively, in presence of chronic VX-770 exposure, indicating that the fraction of CFTR activity correction of these mutants would likely be associated with therapeutic benefit.

## Discussion

Here, we show that, at optimal concentrations, Trikafta corrects the function of maximally activated F508del in homozygous HNE to ~62% of the mean WT-CFTR I_sc_. The magnitude of the I_sc_ in a subset of patients, however, overlaps with some of the I_sc_ in individual WT-HNE, reflecting variability factors that influence the I_sc_ mediated by the phosphorylated WT-CFTR in HNE of non-CF individuals (Supplemental Figure 3). The concentrations of VX-661 and VX-770 used for this study fall within the range of VX-661 plasma concentrations and of VX-770 concentrations in the plasma and nasal tissue of CF patients (34). While the sensitivity of Trikafta corrected F508del-CFTR to endogenous agonists is not known, the achieved correction level, at least in homozygous F508del patients, would be expected to alleviate most of the clinical symptoms in individuals lacking irreversible remodeling of the airway functional and morphological architecture, since heterozygous carriers lack disease symptoms.

The clinical trials with Trikafta showed only partial normalization of the 60%–65% mean baseline ppFEV1 after 24 weeks of treatment in compound heterozygous F508del (16) or at 4 weeks in homozygous F508del patients (15). Similarly, the ppFEV1 gain of 10.6% in compound heterozygous G551D patients after 24 weeks of treatment with ivacaftor resulted in partial normalization of the lung function (41). Long-term studies also indicate that, in spite of the robust ppFEV1 improvement, adult G551D patients still experience progressive loss of their lung function (42, 43) and *P*. *aeruginosa* infection, albeit at reduced frequency (44). The apparent discrepancy between the only partially mitigated CF lung disease in adult patients and the patient-derived epithelial cell data may be accounted for by the long-term effects of chronic lung infection and inflammation, and consequential tissue damage (45). This notion is supported by a recent longitudinal study of ivacaftor therapy in G551D-CFTR patients, which shows the absence of perpetual lung function decline if the therapy was initiated in patients aged younger than 18 years (42). These observations raise the question of whether the magnitude of lung function improvement, particularly in patients with manifest lung disease, can be regarded as sufficient or whether we should strive for further or complementary drug development/treatment and/or earlier onset of treatment for CF patients, requiring decades of therapy.

Viable approaches to further increase the Trikafta therapy efficacy for F508del and rare CFTR mutant correction can be envisioned by implementing (a) triple corrector combinations (21), (b) combining correctors with a CFTR mRNA stabilizer (PTI428) (46) or dual potentiator (47, 48), (c) increasing the constitutive CFTR activity (49), and/or (d) using potentiators that do not to compromise the biogenesis and stability of mutant CFTRs (35, 50). Selection of modulator combinations, however, requires the knowledge of the mechanism of action of individual CFTR modulators.

Combinatorial profiling identified VX-445 as a putative type III corrector, which exhibits a binding affinity of ~80 μM to the isolated F508del–NBD1-1S and suppresses the unfolding trajectory and the aggregation propensity of the mutant domain. The extent of aggregation inhibition of the F508del-NBD1 by VX-445 was similar to that by the bacterial DnaK-DnaJ-GroE chaperone system and to that reported for the Hsc70/Hdj-2 chaperone/cochaperone pair (30), suggesting a direct pharmacochaperone activity of the compound. The increased in vivo potency of VX-445, however, may indicate its intracellular accumulation similar to that observed for VX-770 (36) and/or the exposure of a VX-445 binding surface, which is either modulated by or also constitutes the MSD1/2 and/or NBD2 segments in the context of the full-length F508del-CFTR.

Besides substantially correcting the F508del-CFTR misprocessing, combining VX-445 with type I correctors (e.g., VX-661) also increased the rescue efficacy of rare folding mutants, including S13F, R31C, G85E, E92K, V520F, M1101K, and N1303K, reaching therapeutically relevant correction levels of > 20% of the WT in CFBE41o- cells or HNE. These observations illustrate that the cooperative F508del-CFTR domain misfolding (10, 11, 51) can be robustly reversed by allosteric pharmacochaperones that synergistically stabilize NBD1 unfolding intermediates and the MSD1 by type III (VX-445) and type I (VX-661) correctors, respectively. The VX-661 plus VX-445 also permits allosteric correction of the global conformation defect elicited by other missense CF mutation located in the MSD1, MSD2, NBD1, or NBD2, in agreement with our previous results (21). Thus, it is likely that other folding mutants, including mutant proteins that are resistant to single corrector treatment, will be susceptible to correction with VX-661 plus VX-445 and that establishing mutant responses in patient-derived epithelial cells may lead to the approval of Trikafta for additional CFTR mutations.

## Methods

### CFTR modulators.

CFTR modulators VX-770, VX-661, VX-809, and FDL169 were purchased from Selleckchem. Correctors 4172, 3151 from Life Chemicals, and 6258 from Maybridge have been described before (21). VX-445 EN1 and EN2 were purchased from MedChemExpress, with an enantiomeric excess of 92% and 75.6% as determined by chiral liquid chromatography. ABBV-2222 and VX-445 were synthesized as described below. The combination of VX-661 (lumacaftor), VX-445 (elexacaftor), and VX-770 (ivacaftor) is sold by Vertex Pharmaceuticals under the trade name Trikafta.

### Synthesis of ABBV-2222.

Assembly of ABBV-2222 was accomplished based on variations of previous reports (Supplemental Figure 7) (patent no. US 2016/0120841). In brief, commercially available 2,2-difluorobenzo[d][1,3]dioxole-5-carboxylic acid (structure 1) was reduced with lithium aluminium hydride to form the corresponding alcohol (structure 2), followed by halogenation with thionyl chloride to generate structure 3. Treatment with sodium cyanide afforded the cyano structure 4, followed by base-catalyzed cyclization with 1-bromo-2-chloroethane to form the cyclopropyl group (structure 5). Base treatment transformed the cyano group to the carboxylate, which was then converted to the acyl chloride (structure 6) via thionyl chloride. The aminobenzopyran-like precursor (structure 7), which was sourced commercially, was then coupled with structure 6 using equimolar amounts in the presence of 1 equivalent of base, to finally form ABBV-2222 (structure 8).

### Synthesis of VX-445.

VX-445 was prepared based on a previous report (Supplemental Figure 8) (patent no. WO 2018/107100). An equimolar mixture of 3,3,3-trifluoro-2,2-dimethyl-propan-1-ol (structure 9) and *tert*-butyl 3-hydroxypyrazole-1-carboxylate (structure 10) in toluene was treated with a slight excess of triphenylphosphine and then isopropyl *N*-isopropoxycarbonyliminocarbamate and stirred at 110°C for 18 hours. The solution was then worked up, and structure 11 was purified via flash chromatography, followed by *tert*-butyloxycarbonyl (Boc) removal with 4N HCl to generate structure 12. In a separate reaction, 2,6-dichloropyridine-3-carboxylic acid (structure 13) was *tert*-butylated with Boc anhydride to afford structure 14, which was then reacted with structure 12 under basic conditions to generate the bicyclic structure 15. The *tert*-butyl ester was then cleaved under acidic conditions to form structure 16 and was then activated with carbonyldiimidazole (CDI) in basic conditions to acylate the sulfonamide (structure 17) to form structure 18. Finally, a base catalyzed aromatic substitution reaction was then conducted to incorporate structure 19 into structure 18, followed by flash chromatography to isolate pure VX-445 (structure 20). Structural validation of ABBV-2222 and VX-445 were performed by ^1^H-NMR and liquid chromatography–mass spectrometry (LC-MS).

### Cell lines.

We previously described the generation of CFBE41o- (a gift from D. Gruenert, UCSF, San Francisco, California, USA) cells lines expressing inducible CFTR variants with a 3HA-tag in the fourth extracellular loop (52). New CFTR variants were generated by overlapping PCR mutagenesis–mediated introduction of nucleotide substitutions (10). CFBE41o- cells were grown in MEM medium containing 10% FBS, 10 mM HEPES, and 2 mM L-glutamine (Invitrogen), and the expression of CFTR variants was induced for ≥ 3 days with 500 ng/mL doxycycline (MilliporeSigma).

### HNE.

Tissue was collected by scrape biopsy, and HNE cell isolation was performed as described (53), followed by conditional reprogramming (54). HNE carrying rare CFTR mutations were gifts from W. Finkbeiner (UCSF) or were purchased from the Cystic Fibrosis Canada-Sick Kids Program in Individual CF Therapy. For functional measurements, HNE were seeded at a density of 5 × 10^5^ cell/filter on 1.12 cm^2^ Snapwell filter supports (Corning) and differentiated under ALI by culturing in PneumaCult-ALI medium (Stemcell Technologies) for ≥ 3 weeks.

### CFTR PM density measurement.

The PM density of 3HA-tagged proteins was determined by cell surface ELISA using mouse monoclonal anti-hemagglutinin (HA) antibody (BioLegend, clone 16B12, order no. 901524, 1:2000) (13). Cells were exposed to compounds or 0.2% DMSO (vehicle control) for 24 hours at 37°C in full medium. PM density values were normalized with cell viability measured by alamarBlue Assay (Invitrogen) and related to WT-CFTR by normalizing with CFTR mRNA abundance determined by qPCR as previously (21).

### SPR.

As described in our previous publication (21), binding interactions between biotinylated NBD1 constructs (WT-1S-, F508del-1S-, F508del-3S-NBD1; ~30 kDa each) and corrector compounds were examined with a BIACORE T200 system (GE Healthcare Bio-Sciences AB; Control software v2.0 and Evaluation software v1.0). Briefly, neutravidin (2 mg/mL in water, diluted to 50 μg/mL in 10 mM sodium acetate, pH 5.0; Thermo Fisher Scientific) was amine-coupled to S-series CM5 sensors (~12,000 RU per flow cell) at 25°C using filtered (0.2 μm) and degassed HBS-EP buffer (10 mM HEPES, pH 7.4; 150 mM NaCl; 3 mM EDTA; 0.005% [v/v] Tween-20). After subsequent cooling to 4°C (sample chamber and sensor) and equilibration in PBS running buffer (1× PBS pH 7.4 containing 10% [v/v] glycerol, 2 mM MgCl_2_, 1 mM ATP, 0.05% [v/v] Tween-20), reference (neutravidin only) and active surfaces (100 μg/mL NBD1 constructs in PBS buffer; capture 5000–8000 RU each) were equilibrated in PBS buffer containing 2% (v/v) DMSO at 10 μL/min. The corrector compounds were titrated over reference and NBD1-immobilized surfaces at 25–50 μL/min (1–2 minutes association plus 2–5 minutes dissociation; 2-fold dilution series for all compounds). Between titration series, the surfaces were regenerated at 50 μL/min using two 30-second pulses of solution I (PBS-DMSO buffer containing 1.0 M NaCl and 0.05% [v/v] Empigen [Anatrace]). The rapid, steady-state binding responses (R_eq_) were independent of mass transport limitations and subjected to DMSO solvent correction. All double-referenced data (55) presented are representative of duplicate injections acquired from at least 3 independent trials. To predict overall equilibrium *K_D_* for each NBD1 construct, R_eq_ were averaged near the end of each association phase, plotted as a function of CFTR corrector concentration, and then subjected to nonlinear regression analysis in the T200 evaluation software (“Steady state affinity” model).

### Expression and purification of human CFTR-NBD1 variants and bacterial chaperones.

Recombinant human NBD1 proteins were purified from *E*. *coli* as previously described (18). The NBD1 protein was concentrated to 3–5 mg/mL in buffer containing 150 mM NaCl, 1 mM ATP, 2 mM MgCl_2_, 1 mM TCEP, 10% glycerol, and 50 mM NaPO_4_, pH 7.8. DnaK, DnaJ, and GrpE were expressed and purified as previously described using expression plasmids (pDS56-dnaK-Chis6, pUHE21-2fdD12-dnaJ, pZE2-Pzl-grpE, respectively) and strains (BB1553, W3110), provided by M. Mayer (University Heidelberg, Heidelberg, Germany) (56).

### NBD1 aggregation assay.

The aggregation of human NBD1 variants of CFTR was followed by measuring absorbance at 400 nm in a Tecan Infinite M1000 (Tecan Group) fluorescence plate reader. Absorbance was determined every minutes in 30 μL of F508del–NBD1-1S (6 μM) incubated in 50 mM NaPO_4_, pH 7.5; 150 mM NaCl; 2 mM MgCl_2_; and 1 mM ATP at 32°C in the presence or absence of indicated compounds dissolved in DMSO (57).

### CD spectroscopy.

CD spectroscopy was performed using a Chirascan CD spectrometer (Applied Photophysics). CD scans were acquired between 250 and 195 nm every 1 nm with 0.5 seconds as integration time using a 0.2 mm pathlength cuvette. CD data were obtained for F508del–NBD1-1S at 10 μM in 50 mM sodium phosphate, pH 7.5; 150 mM NaCl; 1 mM ATP; and 2 mM MgCl_2_ at 32°C. Compounds were dissolved in 1,4-Dioxane.

### I_sc_ measurement.

I_sc_ measurement of polarized HNE has been described previously (21, 48). HNE were exposed to compounds or 0.2% DMSO (vehicle control) for 24 hours at 37°C in serum-free PneumaCult-ALI medium (Stemcell Technologies). The transepithelial resistance of differentiated HNE was 423 ± 71 Ω × cm^2^. Cells grown on Snapwell were mounted in Ussing chambers (Physiologic Instruments) in Krebs-bicarbonate Ringer (KBR) buffer (140 mM Na^+^, 120 mM Cl^−^, 5.2 mM K^+^, 25 mM HCO_3_^−^, 2.4 mM HPO_4_, 0.4 mM H_2_PO_4_, 1.2 mM Ca^2+^, 1.2 mM Mg^2+^, 5 mM glucose, pH 7.4), which was mixed by bubbling with carbogen (95% O_2_ and 5% CO_2_). HNE were measured in the presence of basolateral-to-apical chloride gradient, generated by replacing NaCl with 115 mM Na^+^ gluconate in the apical buffer, in the presence of 100 μM amiloride. After compensating for voltage offsets, the transepithelial voltage was clamped at 0 mV (VCC MC8 Multichannel Voltage/Current Clamp, Physiologic Instruments), and current and resistance were recorded at 37°C with the Acquire and Analyze package (Physiologic Instruments).

### Statistics.

Unless otherwise specified, statistical analysis was performed by 1-way ANOVA with post hoc Tukey’s HSD test with the means of at least 3 independent experiments, and the 95% CI was considered significant. Functional measurements in HNE from several homozygous F508del patients were analyzed by paired 2-tailed Student’s *t* test followed by Bonferroni’s FDR correction. Dose-response plots were fitted with a pharmacological dose-response equation using OriginPro 8. Clustering was performed with the Heatmapper software (http://www2.heatmapper.ca/), using the average linkage method and distance calculation by Spearman’s rank correlation.

### Study approval.

The isolation of HNE from healthy and CF human subjects was performed under the protocol and consent form approved by the McGill MUHC Research Ethics Board (MP-37-2018-4089).

## Author contributions

The overall design of the study was contributed by GV and GLL; GV, AR, MAH, and DFDF performed experiments and analyzed the results; MH and TV synthesized and provided ABBV-2222 and VX-445; HX cloned rare CFTR folding mutants and generated cell models; SF and EM collected the patient samples for HNE isolation. The manuscript was primarily written by GV and GLL, with input from all authors.

## Supplementary Material

Supplemental data

## Version 1 08/27/2020

In-Press Preview

## Version 2 09/17/2020

Electronic publication
