# Supplementary material for: Allosteric folding correction of F508del and rare CFTR mutants by elexacaftor-tezacaftor-ivacaftor (Trikafta) combination
Source: JCI Insight. 2020 Sep 17;5(18):e139983. doi: 10.1172/jci.insight.139983 (PMC7526550; doi:10.1172/jci.insight.139983)
Supplement: Supplemental data [file jciinsight-5-139983-s205.pdf]

Supplementary Materials

for

Allosteric folding correction of F508del and rare CFTR mutants by elexacaftor-tezacaftor-ivacaftor (Trikafta) combination

Guido Veit*, Ariel Roldan, Mark A. Hancock, Dillon F. Da Fonte, Haijin Xu, Maytham Hussein, Saul Frenkiel, Elias Matouk, Tony Velkov and Gergely L. Lukacs*.

***Corresponding authors:**

Gergely L. Lukacs
Email: gergely.lukacs@mcgill.ca

and

Guido Veit
Email: guido.veit@mcgill.ca

This file contains:

Supplementary Figures 1-8

Supplementary Table 1

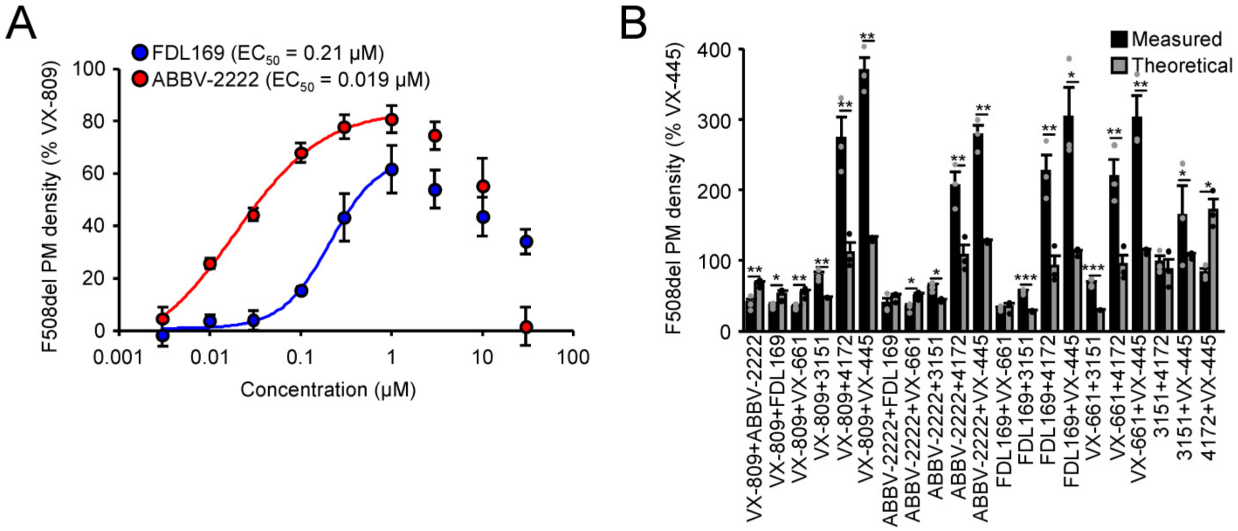

Supplementary Figure 1. Dose response of selected type I correctors and efficacy of corrector pairs for F508del PM density correction in CFBE41o-.

A. Dose-dependent effect (n = 3) of ABBV-2222 and FDL169 on F508del-CFTR PM density in CFBE41o- cells determined by cell surface ELISA and depicted as percentage of VX-809 (3 μM).

B. F508del-CFTR PM density, measured by cell surface ELISA in CFBE41o- cells upon dual or triple corrector exposure (24 h, VX-809 – 3 μM, ABBV-2222 and FDL169 – 1 μM, 3151 and 4172 – 10 μM) in comparison to the calculated additivity of single corrector effects (n = 3). *$P < 0.05$, **$P < 0.01$, ***$P < 0.001$ by unpaired, two-tailed Student's *t*-test to compare between measured and theoretical additivity.

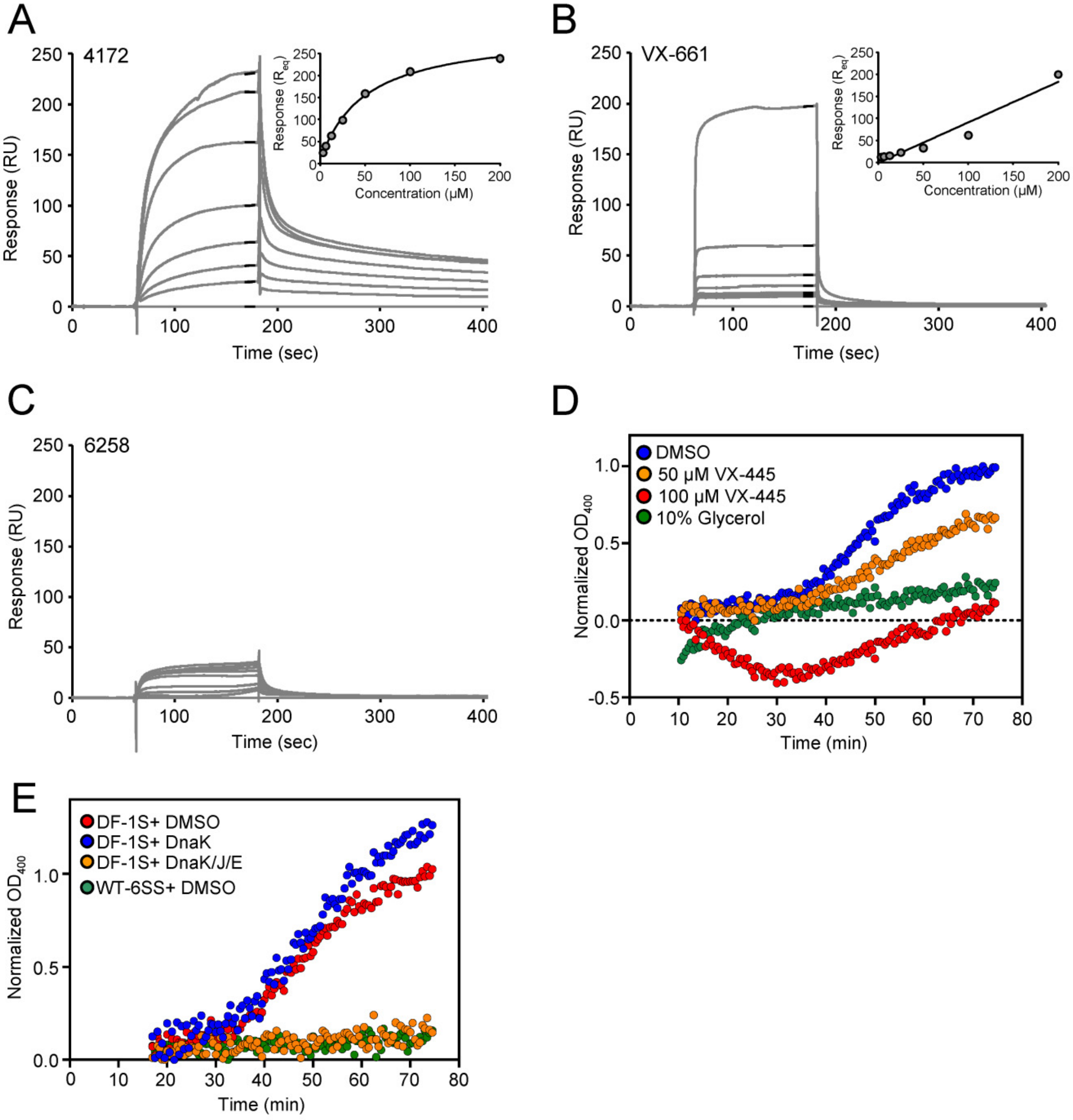

Supplementary Figure 2. Corrector mechanism of action.

A-C. Representative surface plasmon resonance (SPR) sensogram for the binding of 4172 (0-200 µM, A), VX-661 (0-200 µM, B) or 6258 (0-200 µM, C) to immobilized F508del-NBD1-1S. The inserts in A and B show the binding isotherms for 4172 and VX-661, respectively. Curve fitting was performed as described in methods. Traces are representatives of n = 3 independent experiments.

D. Aggregation of F508del-NBD1-1S was followed by turbidity measurement at 400 nm and 32˚C for 70 min in the presence of 1% DMSO, 50 or 100 µM VX-445, or 10% glycerol. Absorbance values were corrected by control experiments performed under the same conditions in the absence of the NBD1 domain and normalized for the maximum amplitude observed for F508del-NBD1-1S in 1% DMSO. Traces are representatives of n = 3 independent experiments.

E. Aggregation of F508del-NBD1-1S in presence of 1% DMSO, 10 µM DnaK, or DnaK-DnaJ-GrpE at 10-2-10 µM, respectively, and of WT-NBD1-6SS in presence of 1% DMSO were followed by turbidity measurement at 400 nm and 32˚C for 70 min. Normalization was performed as described in D. Traces are representatives of n = 3 independent experiments.

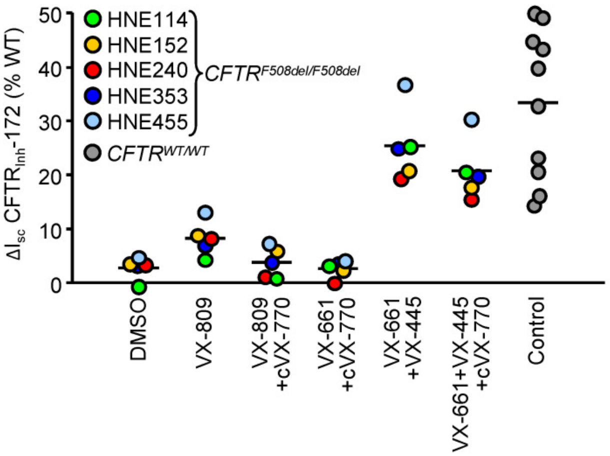

Supplementary Figure 3. Functional correction of F508del-CFTR in human nasal epithelia. Quantification of the $CFTR_{inh}$-172 inhibited current, after stimulation with forskolin (20 µM) and VX-770 (10 µM), in CF-HNE isolated from five different homozygous F508del CF patients after single correctors (VX-809, VX-661 – 3 µM, VX-445 – 2 µM, 24 hours, 37˚C), corrector + chronic potentiator (cVX-770 – 1 µM, 24 hours, 37˚C) or corrector combination as well as WT-CFTR currents in WT-HNE from ten donors. The currents are expressed per cm^2, the same results as percentage of mean WT-CFTR currents are depicted in Fig. 3B.

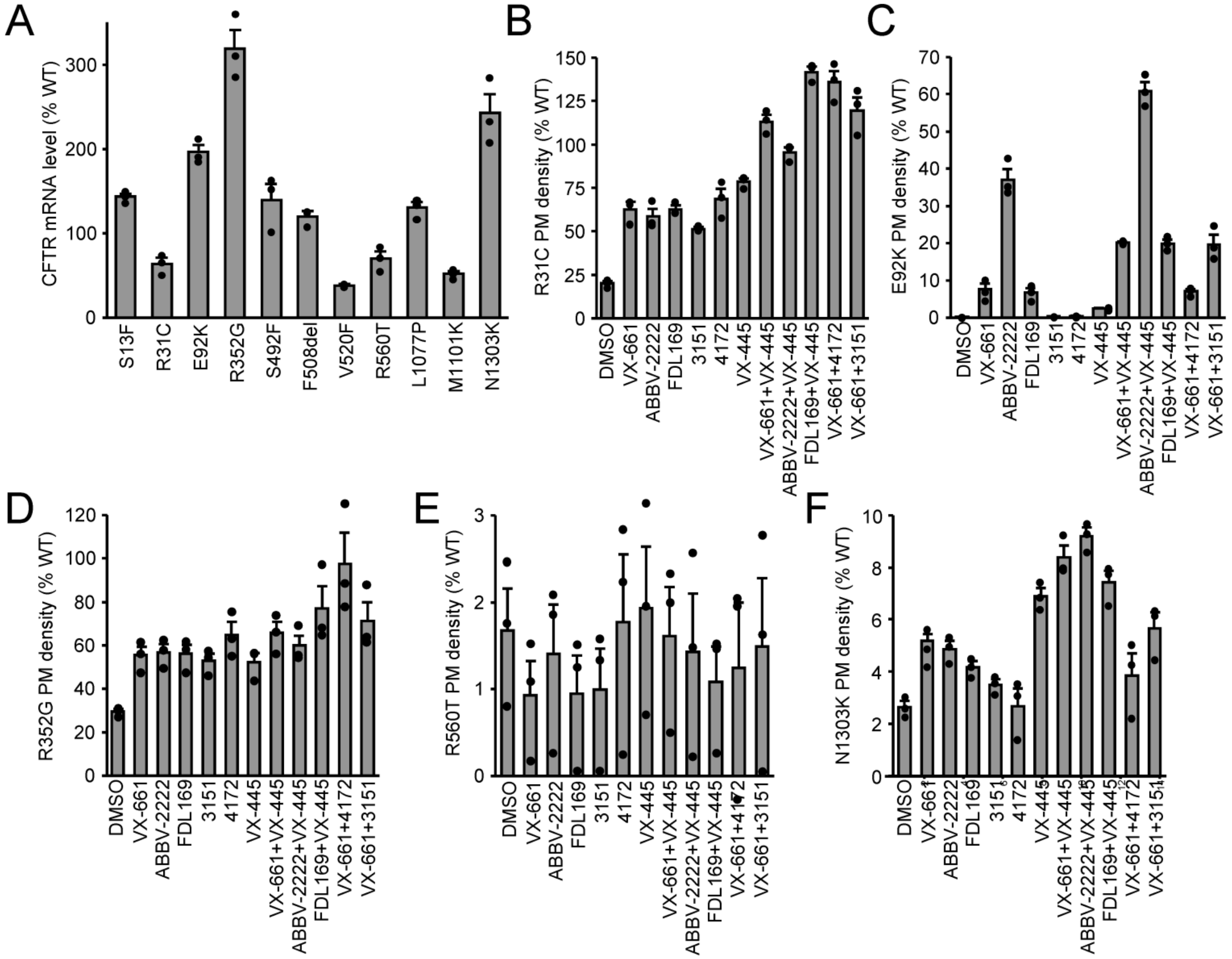

Supplementary Figure 4. Rescue of rare CF folding mutant PM density by dual corrector combinations.

A. Mutant CFTR mRNA expression in CFBE41o- determined by qPCR and expressed as percent of WT-CFTR mRNA level (n = 3).

B-F. PM density of the R31C (B), E92K (C), R352G (D), R560T (E), and N1303K-CFTR (F) alone and after indicated single correctors or corrector combinations treatment expressed as percentage of WT-CFTR in CFBE41o- (n = 3).

Data in A-F are means ± SEM of three independent experiments.

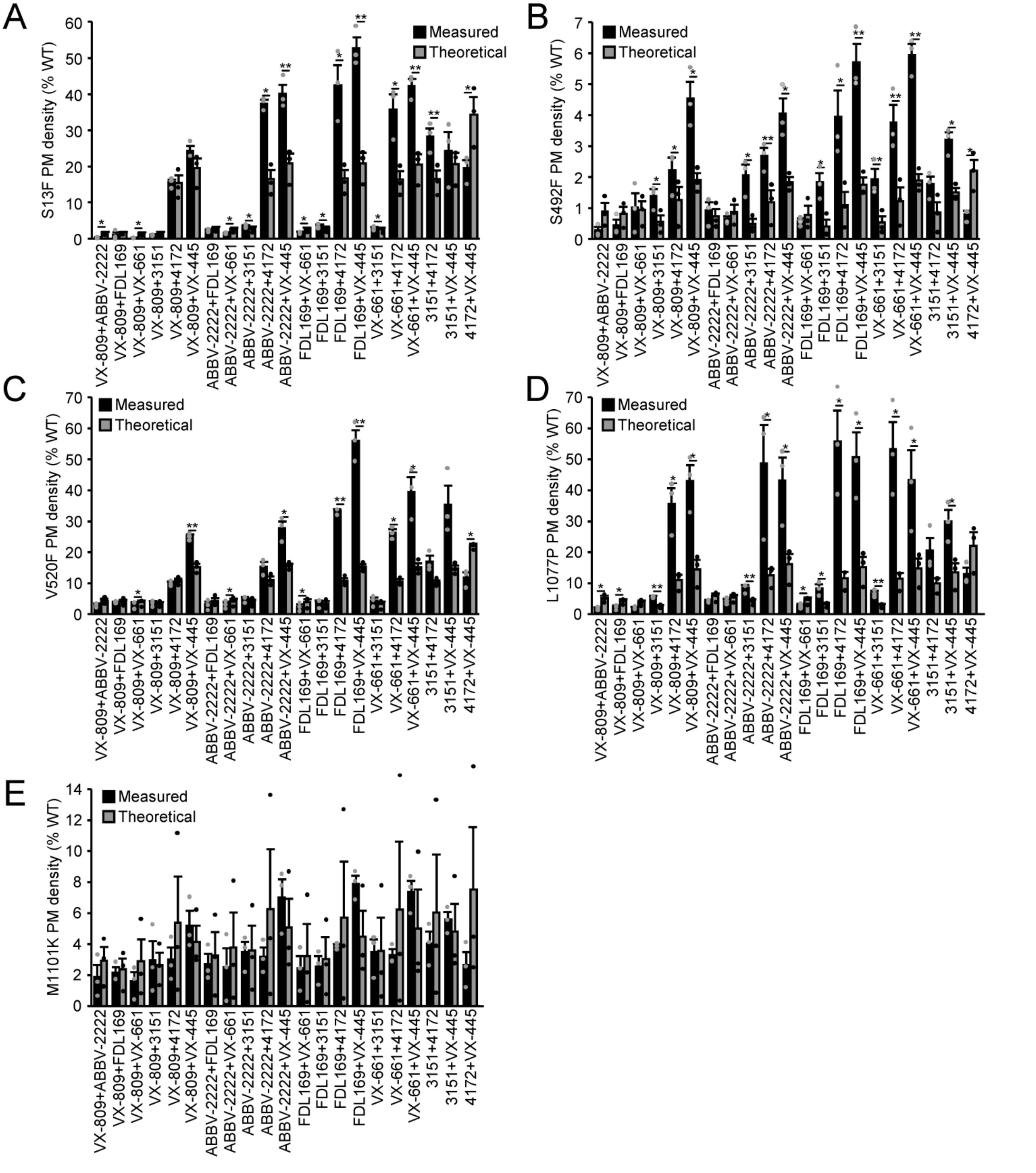

Supplementary Figure 5. The effect of dual corrector combinations on the PM density of CFTR mutants in CFBE41o-.

A-E. Quantification of S13F (A), S492F (B), V520F (C), L1077P (D), or M1101K (E) PM density, measured by cell surface ELISA after dual corrector treatment in comparison to the calculated additivity of single corrector effects (n = 3). The same data, depicted as heat map, is shown in Fig. 4B. Data in A-F are means ± SEM of three independent experiments. **P* < 0.05, ***P* < 0.01by unpaired, two-tailed Student's *t*-test between measured and theoretical additivity.

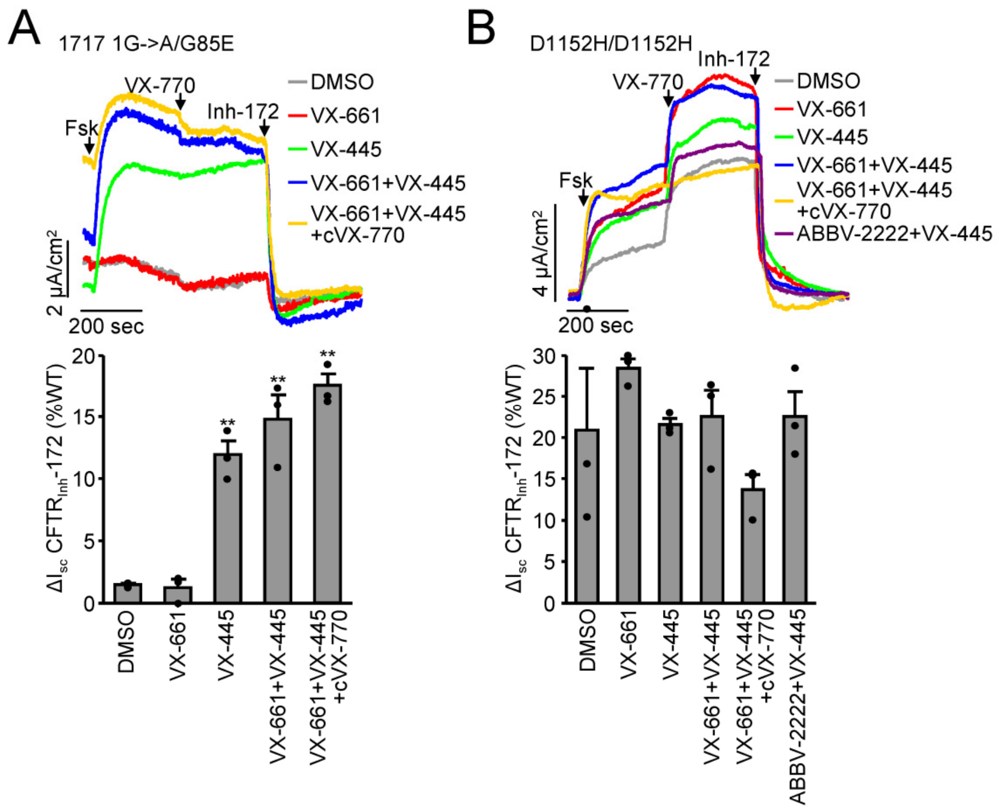

Supplementary Figure 6. Effect of Trikafta on the G85E- and S549R-CFTR function in human nasal epithelia (HNE).

A-B. Effect of indicated single correctors (VX-661, ABBV-2222 – 3 μM, VX-445 – 2 μM, 24 hours), corrector + chronic potentiator (cVX-770 – 1 μM, 24 hours) or corrector combinations on the I_{sc} of HNE with *CFTR*$^{1717-16->A/G85E}$ (A), *CFTR*$^{D1152H/D1152H}$ (B), genotype. Representative traces (top panels) and quantification of the CFTR$_{inh}$-172 inhibited current expressed as percentage of WT-CFTR currents in HNE from ten donors (bottom panels). CFTR-mediated currents were induced by sequential acute addition of forskolin (Fsk, 20 μM) and VX-770 (10 μM) followed by CFTR inhibition with CFTR$_{inh}$-172 (Inh-172, 20 μM) in an intact monolayer with basolateral-to-apical chloride gradient. Data are means ± SEM of three measurements. $^{**}P < 0.01$by one way ANOVA followed by Turkey's post-hoc test.

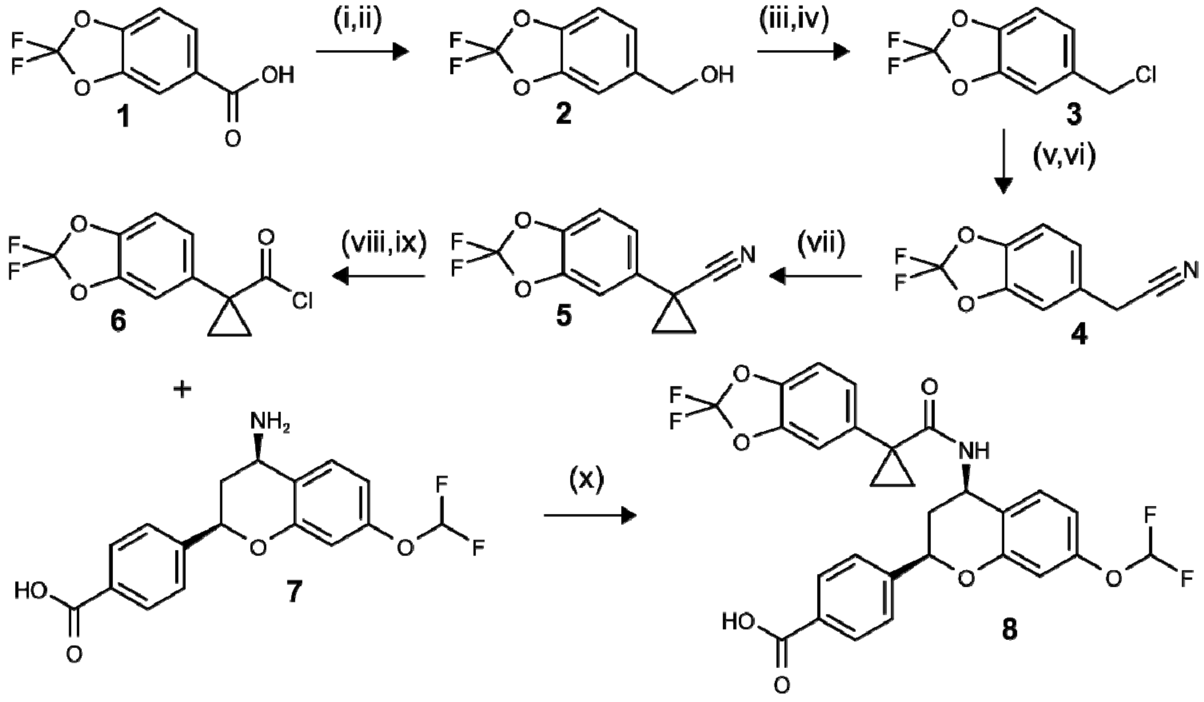

Supplementary Fig 7**. Synthesis of ABBV-2222.**
(i) $LiAlH_4$; **(ii)** NaOH; **(iii)** $SOCl_2$; **(iv)** H_2O; **(v)** NaCN; **(vi)** H_2O; **(vii)** 1-bromo-2-chloroethane, KOH; **(viii)** NaOH; **(ix)** $SOCl_2$; **(x)** DIEA.

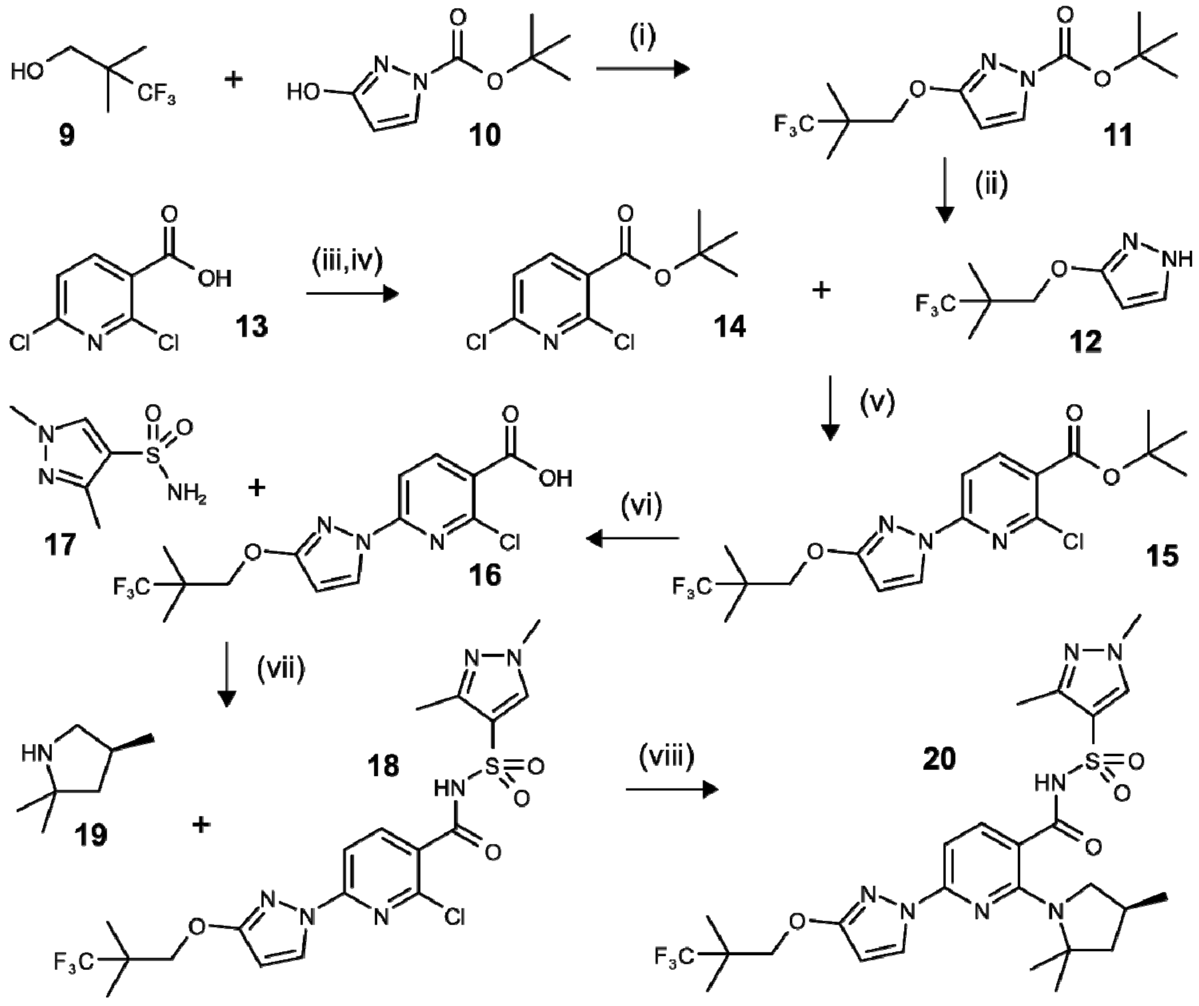

Supplementary Fig 8. Synthesis of VX-445.
(i) PPh_3, isopropyl *N*-isopropoxycarbonyl iminocarbamate, toluene; **(ii)** HCl; **(iii)** Boc_2O; **(iv)** HCl; **(v)** K_2CO_3, DABCO; **(vi)** HCl; **(vii)** CDI, DBU; **(viii)** K_2CO_3.

Supplementary Tables

Supplementary Table 1. Equilibrium dissociation constants (K_D) for corrector compounds binding to CFTR-NBD1 variants, assessed by SPR.

**Compound**	**ΔF508-NBD1-1S Mean K_D ± SEM (μM)**	**ΔF508-NBD1-3S Mean K_D ± SEM (μM)**	**WT-NBD1-1S Mean K_D ± SEM (μM)**
VX-445	82 ± 3 (n = 3)	75 ± 7 (n = 3)	76 ± 7 (n = 3)
4172	40 ± 4 (n =3)	41 ± 5 (n = 3)	47 ± 6 (n = 3)
VX-661	>200 (n = 3)	>200 (n = 3)	>200 (n = 3)
6258	n.b. (n = 3)	n.b. (n = 3)	n.b. (n = 3)

n.b. - no binding
